# Progress in reducing the burden of preterm birth: Are we ready for PRIME time?

**DOI:** 10.1002/pmf2.70210

**Published:** 2026-01-06

**Authors:** Jeffrey L. Ecker

**Affiliations:** ^1^ Department of Obstetrics and Gynecology Massachusetts General Hospital Harvard Medical School Boston Massachusetts USA

**Keywords:** biomarkers, preterm birth, prevention

Tackling the problem of spontaneous preterm birth is a daunting challenge. The associated health, economic, and emotional costs are enormous [1]. The list of promising but ultimately ineffective remedies that have been studied is long [2]. The sensitivity and specificity of tools to identify those at risk are limited [3], and as a result, the complexity of trials and attendant recruitment requires much time and large networks to run. Undaunted, in this issue of *Pregnancy*, Iriye et al. [4] report on efforts to tackle the problem, and though not the final answer, their results offer a dose of hope in our struggle to prevent preterm delivery and suggest promising avenues for further investigation.

The Prematurity Risk Assessment Combined with Clinical Interventions for Improving Neonatal OutcoMEs (PRIME) trial was designed to study the utility of a package of interventions in improving neonatal outcome among a group identified at higher risk for early delivery by the ratio of circulating maternal insulin‐like growth factor‐binding protein 4 to sex hormone‐binding globulin (IGFBP4/SHBG) measured in the second trimester. Each of these proteins, identified through an “omics” approach to discovery, has a biologic link to placental health, and in previous work, an IGFBP4/SHBG predicted delivery at <37 weeks with an area under the receiver operator curve of 0.75 and odds ratio of 5.05 for a sensitivity of 0.75 and a specificity of 0.74 [5]. The PRIME consortium enrolled individuals with singleton pregnancies and excluded potential subjects with a history of spontaneous preterm birth or a short cervix (<25 mm) identified by second‐trimester ultrasound, choosing these exclusions presumably because treatment of such patients to prevent preterm birth was felt to be standard of care (PRIME recruitment began in 2020 before approval for intramuscular (IM) progesterone was withdrawn). Those recruited were randomized 1:1 to either a usual care arm, in which neither patients nor those caring for them were aware of IGFBP4/SHBG ratios, or a guided care arm, in which the 15% with the highest ratios were approached for consent to be treated with a package of interventions that included vaginal progesterone, the addition of low‐dose aspirin for those who had not otherwise already been prescribed aspirin prior to enrollment, and regular, scripted, weekly phone check‐ins with study nurses. (The intervention protocol also included two additional ultrasounds to measure cervical length with the offer of cerclage for anyone who developed a short cervix, but among sixteen with a subsequent short measurement, no cerclages were placed.) Prespecified primary outcomes were a composite of neonatal morbidity and mortality and neonatal length of stay.

Enrollment for PRIME was halted early by the study's data and safety monitoring board (DSMB) when interim analysis demonstrated that a reduction in neonatal length of stay had crossed a predesignated efficacy threshold. All enrolled to that point were followed through delivery and had their neonatal outcomes collected, and then were analyzed in an intention‐to‐treat analysis, including all with available delivery data. This analysis demonstrated a significant reduction in composite neonatal morbidity (odds ratio, 0.80; 95% confidence interval [CI], 0.66–0.96) and neonatal length of stay (incidence rate ratio, 0.95; 95% CI, 0.92–0.98), the latter driven by fewer neonatal intensive care unit (NICU) admissions, as lengths of NICU stays were similar in each arm. Average gestational age at delivery and birthweight were not different between the two groups, results that the authors had anticipated and ascribed to limitations of power and the overwhelming majority of patients in each arm who, as expected, delivered at term. They noted a shift toward more advanced gestational ages among those who did deliver <37 weeks in the intervention arm, however, and this trend, as well as a matching one toward higher birthweight, is clearly seen in the published forest plot. The authors calculated a number needed to screen (NNS) of 38.2 and 4 to reduce one NICU admission and one NICU day, respectively; NNSs that the authors argue compare quite favorably with that estimated for transvaginal evaluation of cervical length to prevent one preterm birth. Only 58% of patients in the guided care arm with a top‐tier IGFBP4/SHBG ratio consented to the study interventions, and, anticipating the possibility of such dropout, the investigators prespecified a modified intention‐to‐treat analysis excluding those who declined the intervention bundle in the guided care group and any in either arm who were diagnosed with COVID‐19 during hospital evaluation after randomization, given the association of such infections with adverse outcomes. Not surprisingly, the modified analysis showed a more pronounced effect from the package in reducing neonatal morbidity and mortality and length of stay. In an interesting and salient observation, albeit an anecdotal one, the authors note that no patient in the open‐label arm who accepted intervention delivered at less than 32 weeks. Taken altogether, this analysis picture offers much that is positive and promising.

In all studies with rich data sets like this one, there are inevitably some analyses that readers wish had been included but were not. Here, that list starts with a wish for the utility of the IGFBP4/SHBG ratio in predicting preterm delivery in the study population. Was the ratio predictive and with the expected sensitivity and specificity? Analysis of the usual care group would have been informative in this regard and helpful. The authors highlight possible confounding as a limitation of their open‐label study design that made patients and providers aware of who in the guided care arm was at higher risk for premature birth. Informed by this news, could providers or patients have taken additional steps, separate from the studied bundle, that might have in some way positively affected outcomes? Comparing outcomes between groups in the open‐label arm who did or did not accept the bundle would have shed at least some light on this possibility. The study's discussion suggests that IGFBP4/SHBG may have utility to “inform strategies to reduce events related to preterm birth,” but those events in the PRIME study that prompted preterm delivery and their distribution are only briefly listed with little consideration. Understanding more regarding the rates of preterm labor or rupture of membranes, as well as maternal or fetal indications for planned early delivery, would be revealing and could potentially help refine the design of both future studies and care. It is also natural to wonder which of the three components that made up the intervention bundle had the biggest impact on the outcome. Analysis of compliance data, at least some of which seems to have been part of the weekly nursing check‐ins, might offer important detail, as would a deeper dive to determine which patient and pregnancy characteristics were associated with key outcomes. Exploring these questions and many others should be the target of future secondary analyses, many of which I suspect the authors already are planning.

While the magnitude of the intervention's effect suggested by odds and incidence ratios and their associated CIs in the PRIME study may seem modest, a modest relative improvement of a prevalent driver of health such as preterm birth may be important and impactful when judged in absolute terms. A reduction in annual health care costs related to preterm birth of “just” 20%, for example, would total more than two billion dollars annually in the United States alone. Accordingly, the positive outcome from this large randomized controlled trial (RCT) understandably raises the question of whether screening with IGFBP4/SHBG and deploying the package of interventions studied by Iriye et al. are something ready for general implementation. If not, what should further studies look like and how might those be best undertaken? Other than describing IGBP4/SHBG as having a “potential role” and the improvement found as “measurable,” the paper's discussion unfortunately does not include the investigators’ answers to those two key questions, but for many reasons, I believe that is not time, at least not yet, to bring the PRIME protocol online as part of day‐to‐day clinical practice. Though the principal outcomes showed a statistically significant difference, their associated CIs come close to crossing one, and with just a very few premature deliveries more in the guided arm or fewer in the usual care arm, this would have been a negative study. Curiously, in this regard, although the DSMB stopped enrollment when interim analysis demonstrated a hazard ratio for neonatal length of stay of 0.49, when final data were collected and analyzed for all those enrolled, the calculated incident rate ratio was 0.94: no small shift toward the null. As well and as already discussed, the possibility of confounding in any study that forgoes placebo controls cannot be discounted. Though the investigators hypothesized that combining therapies may increase overall efficacy, enthusiasm for immediate adoption of their bundle is also tempered by limited evidence that any of the bundle's component parts, when used alone, are efficacious in reducing spontaneous preterm birth in individuals with normal cervical length [6, 7]. Finally, the planned cost and cost‐effectiveness analysis of PRIME is still to be done, and committing to any intervention before better understanding the balance of cost to benefit seems imprudent, if not irresponsible.

Although it may not be time for wide clinical implementation, it is time to organize next studies whose data, if similarly encouraging, will further the case for adoption. What might next studies look like? They might start by consenting all patients from the start to biomarker measurement and randomization to arms that will include placebo controls for all aspects of the studied package. The authors avoided placebos in this first trial, concerned that requiring participants in all arms, regardless of their risk for preterm birth, to take pills by mouth and place others vaginally would challenge recruitment. Indeed, a significant portion of those identified at increased risk in the open‐label/guided care arm declined the study's intervention when second, specific consent was asked. Advertising this aspect of the study up front and making it a requirement for participation might still be a bar too high for some who are approached, but doing so would hopefully decrease drop‐out later. Now that approval for IM progesterone has been withdrawn and there is no standard intervention in the next pregnancy for those with a history of preterm labor or preterm rupture of membranes, there should be no objection to including such patients in the next study of IGFBP4/SHBG guided care, and one imagines those patients would be motivated to enroll. Recruitment for large RCTs is always challenging, however, and we should commit individually as obstetric care providers and researchers and more broadly as a specialty and subspecialty to doing all we can to support further study, given the central importance this work has to our field and for our patients.

What will future study show? There are many reasons to be encouraged by this report from the PRIME group, and the authors may be correct in proposing a multipronged approach to a problem that, though homogenous in outcome, is likely the end point of heterogeneous paths and prompts. It may also be that these serum analytes, directed at targeting variation in placental health and biology, identify a group of pregnancies particularly amenable to treatment. Yet if we've learned nothing else from past adventures in treating those at risk for preterm delivery, it is that first enthusiasm is not a substitute for further study. The history of preterm birth prevention includes many examples of interventions implemented with great effort after early studies offered promising results, only to have follow‐up data and analysis prove it all a bust. Our experience with IM progesterone, home uterine activity monitoring, terbutaline pumps, screening and treating for bacterial vaginosis, and many other fixes for preterm birth—each framed by reasonable hypotheses and supported by at least some encouraging data—has taught us that we must carefully replicate and thoughtfully validate before going all in.

## CONFLICT OF INTEREST STATEMENT

The author declares no conflicts of interest.

## FUNDING INFORMATION

The author received no specific funding for this work.
